# Assessing the knowledge, attitude, and practices of reproductive health schemes among tribal women in Jharkhand: A cross-sectional study

**DOI:** 10.1186/s12889-026-28411-z

**Published:** 2026-07-08

**Authors:** Rohit Raj, Jarina Begum, Syed Irfan Ali, Anushka Priya Sharan, Shaik Ahammad Basha, Abhishek Kumar, Swati Shikha

**Affiliations:** Department of Community Medicine, Manipal Tata Medical College, Manipal Academy of Higher Education, Manipal, Karnataka India

**Keywords:** Reproductive health schemes, Tribal women, Healthcare accessibility, Maternal health disparities, Healthcare utilisation

## Abstract

**Background:**

Women of reproductive age form a significant population in India, with Jharkhand’s tribal communities facing acute maternal health challenges. Despite progress in reducing maternal mortality, service utilisation remains low, with high rates of anaemia and undernutrition. This study aims to assess the knowledge, attitude, and practices of healthcare schemes among tribal women in the reproductive age group.

**Methods:**

A cross-sectional study was conducted in East Singhbhum, Jharkhand (March-November 2024), using purposive, simple random, and stratified sampling. Two blocks and twelve villages were selected, with participants divided into early adolescents (10–14 years), late adolescents (15–19 years), and women (20–49 years) to avoid overlap. The final sample size was 745. Analysis employed descriptive statistics and chi-square tests. Knowledge, Attitude, and Practices (KAP) scores regarding eight reproductive health schemes were also analysed.

**Results:**

Most tribal women (ages 20–29) were married housewives with limited education, while adolescent girls were primarily students. Among women (20-29years), 76.6% had partial awareness of health schemes; only 1.7% were fully informed. About 62.3% accessed benefits, and 55% appreciated incentives. Adolescents (10-14years) showed 72.8% partial awareness; the 15–19 group had 93% awareness and 86% utilization. Education and tribal identity significantly influenced KAP outcomes (*p* < 0.001), underscoring schooling’s critical role.

**Conclusion:**

The study reveals notable gaps in knowledge, attitudes, and practices regarding healthcare schemes among tribal women and adolescent girls. KAP scores highlighted knowledge deficits, and the majority had a positive attitude. However, the practices related to the utilisation of reproductive health schemes were found to be more inclined towards the schemes with cash incentives.

**Trial Registration:**

Clinical Trial Registry India (CTRI) CTRI/2024/03/063824 [Registered on 08/03/2024].

**Supplementary Information:**

The online version contains supplementary material available at https://doi.org/10.1186/s12889-026-28411-z.

## What is already known on this topic

*Previous research indicates low awareness and limited use of reproductive health schemes among tribal populations in India*,* influenced by education*,* socio-economic conditions*,* and cultural factors. However*,* data on adolescent girls and tribal women within a multi-level analytical framework remains scarce.*

## What this study adds

*This study offers an in-depth assessment of reproductive health scheme-related knowledge*,* attitudes*,* and practices among tribal women and adolescent girls using the Socio-Ecological Model. It emphasises education*,* tribal identity*,* and age as key influencers*,* while exposing barriers like eligibility issues and bureaucratic delays. It advocates for culturally sensitive outreach and streamlined administration to enhance utilisation and health outcomes in tribal areas.*

## How this study might affect research, practice or policy

*This study highlights the need for culturally sensitive*,* targeted strategies to improve awareness and use of reproductive health schemes among tribal populations. It advocates for integrating reproductive health education in schools and enhancing frontline workers’ roles. Policymakers can apply these insights to streamline implementation*,* minimise administrative hurdles*,* and develop inclusive*,* context-specific healthcare interventions.*

## Background

Women in the reproductive age (15–49 years) account for approximately 70% of the population in developing countries. In India, they represent about 22.2% of the total population [1]. India records approximately 27 million births annually, with a large number of women experiencing pregnancy-related complications. Each year, more than 57,000 maternal deaths are reported, with rural regions witnessing higher maternal mortality rates. This study focuses on the state of Jharkhand, located in the east-central region of India, with rich biodiversity and a sizable tribal population [2]. The scheduled tribe population is around 8.6 million, with the majority living in rural areas [2]. These communities face several health challenges due to economic hardship, limited healthcare access, and strong reliance on traditional practices. Major health concerns in this region include maternal and child health problems, malnutrition, anaemia, inadequate medical infrastructure, and cultural barriers to modern healthcare [3]. However, the state has made notable progress in reducing maternal mortality, achieving a Maternal Mortality Ratio (MMR) of 56 per 100,000 live births between 2018 and 2020, which is significantly lower than the national average of 97. Despite this improvement, the state’s utilisation of maternal health services remains suboptimal. According to the National Family Health Survey (NFHS-5) conducted between 2019 and 2021, only 36.4% of mothers in Jharkhand had at least four antenatal care (ANC) visits, compared to the national average of 54.2% [4]. Around 14.9% of mothers took Iron-Folic Acid (IFA) supplements for 180 days or more during pregnancy. According to the NFHS-5 (2019-21) report for Jharkhand, anaemia prevalence among pregnant women (15–49 years) was 56.8%, and 65.3% among adolescent girls (15–19 years), higher than the national averages of 52.2% and 60.2%, respectively. Furthermore, 26.2% of women in Jharkhand had a Body Mass Index (BMI) below 18.5 kg/m², indicating undernutrition compared to the national rate of 18.7%. Regarding family planning in rural Jharkhand, only 0.3% underwent male sterilisation, while 0.4% underwent female sterilisation; use of condoms and oral pills was 3.5% and 3.1%, respectively [4].

These disparities suggest that there is a need to enhance the accessibility and uptake of maternal health services in Jharkhand to ensure sustained improvements in maternal health outcomes. To address these disparities, the Indian government has introduced multiple health programs all over India, aimed at improving the well-being of women in the reproductive age group. India’s public healthcare is structured across primary (Sub-Centres, PHCs), secondary (CHCs), and tertiary (district/teaching hospitals) levels, delivering largely tax-funded services accessible to all. Initiatives such as the Janani Suraksha Yojana (JSY) [5], Janani Shishu Suraksha Karyakram (JSSK) [6], Pradhan Mantri Matru Vandana Yojana (PMMVY) [7], Pradhan Mantri Surakshit Matritva Abhiyan (PMSMA) [8], Rashtriya Kishor Swasthya Karyakram (RKSK) [9], Poshan Abhiyaan (PA) [10], Anemia Mukt Bharat (AMB) [11], and schemes under the National Family Welfare Program-including the Accredited Social Health Activist (ASHA) Scheme [12] and sterilization compensation schemes [12] are all geared toward this objective. This study aims to evaluate the knowledge, attitudes, and practices of these health programs among tribal women in the reproductive age group.

## Methodology

A cross-sectional study was conducted between March 15 and November 10, 2024, in East Singhbhum district of Jharkhand, India. This district comprises two subdivisions- Dalbhum and Ghatsila, covering 11 administrative blocks. First, purposive sampling was conducted among tribal communities in a specific region, East Singhbhum district in Jharkhand, India.

**Fig. 1:**
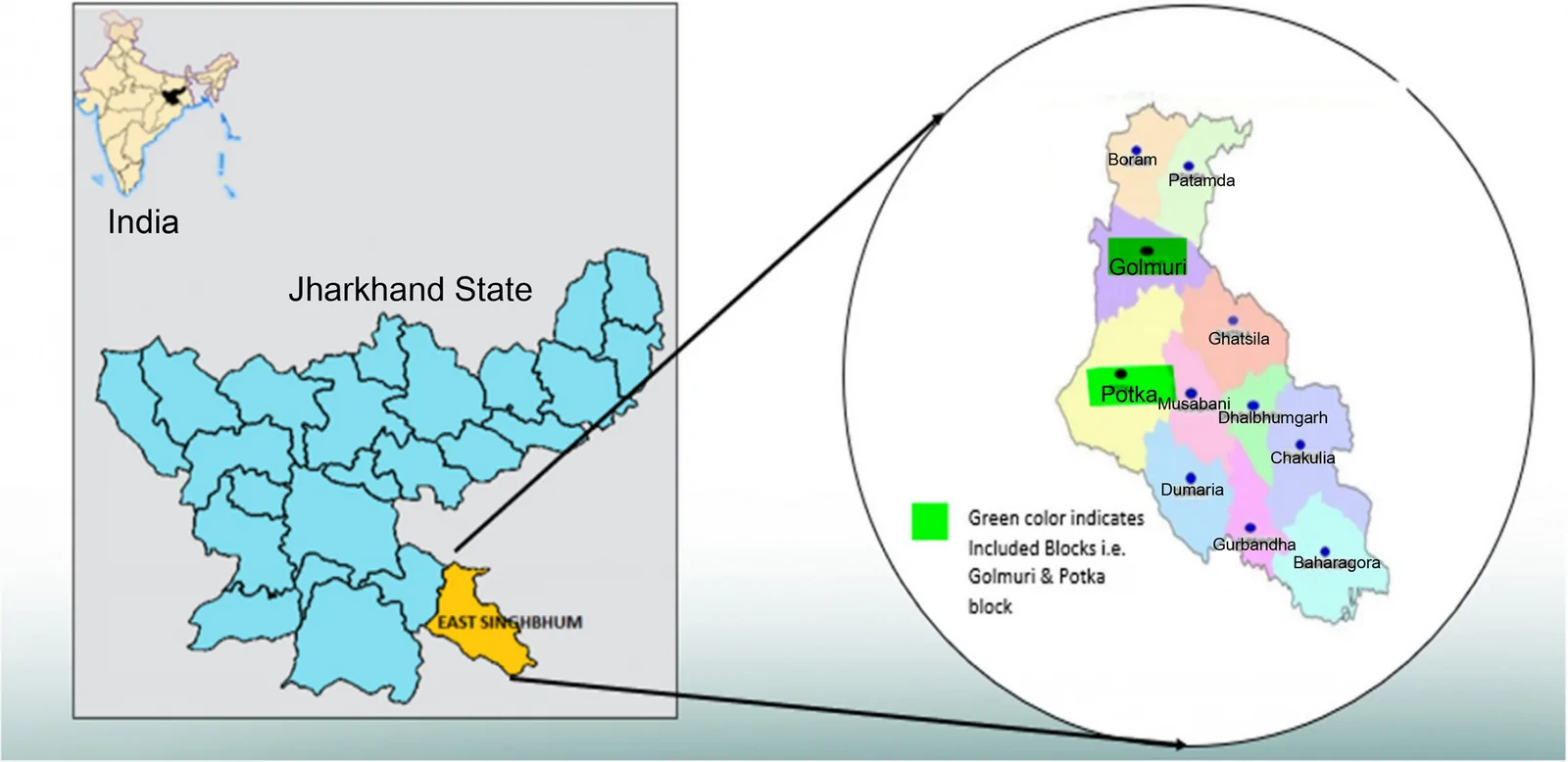
Map of East Singhbhum district, state Jharkland, India, with the study area. Golmuri and Potka block highlighted with green color

Simple random and stratified sampling techniques were used by the lottery method. Two blocks, Golmuri cum Jugsalai and Potka, were chosen at random; six villages from each block (12 in total) were included. Each village was stratified into two strata: the tribal adolescent girl group and the tribal women of reproductive age group. Figure 1 depicts the study area within East Singhbhum district, Jharkhand, India.

The required sample size was calculated using an online tool (calculator.net) [13], based on a 95% confidence interval, 5% margin of error, 50% population proportion, and a total population of 258,454. Using the formula N = (Z1-α/2) ² * p(1-p) / d², the base sample size was 385. Adjusting for a 20% non-response rate and a 1.5 design effect, the final sample size was rounded to 745. Hence, per village, tribal adolescent girls (*n* = 32) and tribal women of the reproductive age group (*n* = 30) were included. This number division was based on preliminary research among (*N* = 100) from one village, which showed an almost equal split between adolescent girls 10–19 yrs (55%) and reproductive-age tribal women 15–49 years (45%). To emphasise tribal women’s reproductive health insights while ensuring adolescent comparison, we included approximately 30 tribal women and 32 adolescent girls per village. The actual non-response reported during the data collection was 2%. The adolescent age group is from 10 to 19 years, and reproductive age women are from 15 to 49 years. To eliminate any overlap, for 10–19 years, we divided participants into two mutually exclusive categories: group 1: early adolescents (10–14 years) and group 2: late adolescents (15–19 years). Similarly, for the 15–49 years group, particularly for participants aged 15–19 years, we divided participants into two mutually exclusive categories: group 1: late adolescents (15–19 years), and group 2: women (20–49 years). While doing the statistical analysis, we have finally three groups to eliminate any overlap, particularly for participants aged 15–19 years, we divided participants into three mutually exclusive categories: group 1: early adolescents (10–14 years), group 2: late adolescents (15–19 years), and group 3: women (20–49 years). Each participant belongs exclusively to one group, ensuring that no individual is counted more than once and facilitating clarity in data collection and analysis.

Only individuals belonging to tribal communities who voluntarily consented to participate were included. Exclusion criteria involved those who had migrated, were lost to follow-up, or had chronic illnesses such as diabetes, hypertension, cardiovascular diseases, thyroid disorders, tuberculosis, other respiratory conditions, or cancer.

Data collection was carried out using a semi-structured validated questionnaire and was made available as a supplementary file in a separate publication [4]. This tool comprised two sections: Section A collected demographic details, while Section B focused on a baseline survey assessing the Knowledge, Attitudes, and Practices (KAP) of tribal women regarding eight key reproductive health schemes in India mentioned in Table 1. Eight healthcare schemes in India included in the study.

**Table 1:** Eight Healthcare schemes in India included in the study

Adolescent Girls schemes	Schemes for the reproductive age group of women
1. Rashtriya Kishori Swasthya Karyakram (RKSK) [9]	4. Janani Suraksha Yojana (JSY) [5]
2. POSHAN Abhiyan (PA) [10]	5. Janani Sishu Suraksha Karyakaram (JSSK) [6]
3.Anaemia Mukt Bharat (AMB) [11]	6.Pradhan Mantri Matru Vandana Yojana (PMMVY) [7]
	7. Pradhan Mantri Surakshit Matritva Abhiyan (PMMVY) [8]
	8. Family Planning Schemes under NHM, including [12]: a. ASHA Scheme b. Enhanced Compensatory Scheme
	8. Anemia Mukt Bharat (AMB) [11]

The data collection questionnaire was designed to measure demographic information and the knowledge, attitudes, and practices of tribal women towards reproductive health schemes. It comprised a total of 28 questions with closed and open-ended questions. Out of which six were demographic questions, six were knowledge-based questions, five were attitude-related questions, and eleven were practice-oriented questions. The instrument was self-developed and subjected to content validation, where it realised a Content Validity Index (CVI) value of 0.924. Cronbach’s alpha, which measures reliability, was calculated using the formula: α = (K / (K-1)) * ((Sy² - ΣSi²) / Sy²). Where K is the number of questions, Sy² stands for variance, and ΣSi² represents the sum of the variances. The total Cronbach’s alpha value realised was 0.7374, representing acceptable internal consistency.

Analysis of data entailed the computation of frequencies, percentages, and proportions. The chi-square test was used for categorical data, and the KAP score was assessed. Statistical analysis was all done using Jamovi software (Version Solid 2.2.5). The open-ended questions were analysed manually by two independent researchers, and any discrepancies were resolved by discussion between the researchers. Based on the responses from participants, codes were developed, and themes were generated. For maintaining the transferability of the findings, participants’ responses were presented as it is unaltered. Various themes developed were partial awareness, cash incentives, source of information, and participant recommendation.

### Operational definition

#### Not aware

A participant is considered partially aware of the specific reproductive health scheme if they are not able to identify even a single component of the services mentioned under the healthcare schemes.

#### Aware

A participant is considered fully aware of the specific reproductive health scheme if they are able to identify all the components of the services mentioned under the healthcare schemes.

#### Partially aware

A participant is considered partially aware of the specific reproductive health scheme if they are able to identify one or more but not all components of the services mentioned under the healthcare scheme.

#### Comprehensive knowledge

Refers broadly to a level of understanding of a fully aware individual who correctly identifies all the components of the services mentioned under the reproductive health care schemes.

#### Patient and public involvement statement

The study engaged tribal communities by taking permission from the State tribal welfare officer, Civil surgeon (senior doctor cum chief medical officer), village gatekeepers such as village heads and health workers to ensure cultural appropriateness. The study adhered to the ethical principles outlined in the World Medical Association’s Declaration of Helsinki for research involving human subjects [14]. Written informed consent was obtained from all participants, with support from community volunteers during data collection. Future efforts aim to co-develop awareness strategies with the community based on these insights.

## Results

The socio-demographic profile indicates that most tribal women were aged between 20 and 29 years, predominantly belonging to the Santhal and Bhumij tribes. A majority were married and housewives, with a considerable number having no formal education. In contrast, most adolescent girls were students; those aged 10–14 were mainly in upper primary school, while those aged 15–19 years were in higher secondary education. Complete demographic details are available in Table 2.

**Table 2:** Socio-demographic status of the three groups

Variable	Category	Tribal Women (20–49 years) *n* = 355	Adolescent Girls (10–14 years) *n* = 349	Adolescent Girls/Women (15–19) *n* = 43
Age	10–14 Years	**-**	349 (100%)	**-**
	15–19 Years	**-**	**-**	43 (100%)
	20–29 Years	184 (51.8%)	**-**	**-**
	30–39 Years	77 (21.7%)	**-**	**-**
	40–49 Years	94 (26.5%	**-**	**-**
Religion	Sarna Dharam	355 (100%)	349 (100%)	43 (100%)
Tribal Affiliation	Santhal	141 (39.7%)	170 (48.7%)	16 (37.2%)
	Munda	17 (4.8%)	12 (3.4%)	2 (4.7%)
	Bhumij	119 (33.5%)	102 (29.2%)	14 (32.6%)
	Ho	62 (17.5%)	52 (14.9%)	11 (25.6%)
	Sabar	16 (4.5%)	13 (3.7%)	-
Education level	No formal education	104 (29.3%)	2 (0.6%)	1 (2.3%)
	Primary education (Class 1–5)	41 (11.6%)	120 (34.5%)	-
	Upper primary education (Class 6–8)	71 (20%)	227 (65.5%)	3 (7.0%)
	Secondary education (Class 9–10)	93 (26.2%)	-	11 (25.6%)
	Higher secondary (Class 11–12)	36 (10.1%)	-	26 (60.5%)
	Graduation & above	10 (2.9%)	-	2 (4.6%)
Occupation	Housewife	316 (89.0%)	-	5 (11.6%)
	Self-employed	15 (4.2%)	-	-
	Student	3 (0.8%)	348 (99.7%)	38 (88.4%)
	Homemaker	10 (2.8%)	1 (0.3%)	-
	Government Job	4 (1.1%)	-	-
	Daily wage worker	6 (1.7%)	-	-
	Private Job	1 (0.3%)	-	-
Marital Status	Married	339 (95.5%)	-	5 (11.6%)
	Unmarried	4 (1.1%)	349 (100%)	38 (88.4%)
	Widowed	12 (3.4%)	-	-

The KAP analysis revealed differences in awareness and utilisation of healthcare schemes. Among tribal women (*n* = 355), 76.6% (271) had partial awareness, 23.4% (84) were unaware, and only 1.7% (6) had comprehensive knowledge. While 49.9% (177) believed schemes improved healthcare, 55.2% (196) valued incentives, and 54.9% (195) endorsed community involvement, only 62.3% (221) accessed benefits, 36.6% (130) received financial support, and 63.6% (225) were unsure of scheme adequacy. Among younger adolescent girls 10–14 years (*n* = 349), 72.8% (254) were partially aware, with only 0.3% (1) fully informed. In the 15–19 age group (*n* = 43), awareness and utilisation were higher, with 93.0% (40) having some knowledge and 86.0% (37) accessing benefits.

Most of the participants were aware of the schemes; their knowledge was often partial, with a strong inclination toward those offering cash benefits. The primary sources of information for women were the community health actors, especially Accredited Social Health Activist (ASHA), who connect rural women to maternal and child health services, and Anganwadi Workers (AWWs), who focus on nutrition and early childhood care, are pivotal despite being volunteers paid via modest incentives, while adolescent girls received information mainly through schools.

Attitudinal responses were generally positive, with most respondents agreeing on the importance of healthcare schemes, incentives, and community participation. However, practices showed mixed results, while many availed benefits, a substantial proportion either did not or were unsure about them. Common barriers to utilisation included lack of awareness, eligibility issues (such as absence of bank accounts), and distrust in the healthcare system due to procedural delays. Participants recommended improving awareness through local language communication, pictorial representations, and community engagement methods like street plays.

The evaluation of KAP concerning reproductive health schemes among adolescent girls aged 10–14 revealed strong associations with factors such as education, tribal background, and occupation. Educational level (*p* < 0.001) and tribal identity (*p* < 0.001) emerged as key determinants of awareness, emphasising the critical role of formal education in enhancing understanding. Attitudes were measured based on the Likert scale questions, such as Strongly disagree (score 1) to Strongly agree (score 5). It was found that attitude was significantly shaped by education, influencing views on the benefits of healthcare schemes (*p* < 0.001), financial incentives (*p* < 0.001), and the importance of community participation (*p* < 0.001). Tribal affiliation significantly affected attitudes toward incentives (*p* < 0.001) and overall scheme experience (*p* = 0.032). Practice-wise, scheme use was notably linked to both education (*p* < 0.001) and tribal identity (*p* = 0.040), with access to financial benefits also tied to tribal background (*p* < 0.001). Among older adolescent girls (15–19), awareness was significantly influenced by tribal identity (*p* = 0.008) and occupation (*p* < 0.001), while knowledge of services was associated with occupation and marital status (*p* = 0.018). For tribal women aged 20–49, both education and tribal identity played important roles in shaping awareness and attitudes.

The KAP score was determined through participants’ responses to categorised questions under KAP. Each participant group-tribal women (15–49 years), adolescent girls (10–14 years), and a mixed group (15–19 years)-was given a designated number of questions per category. The scores were summarised using statistical measures such as mean, standard deviation, median, and Interquartile Range (IQR). The overall KAP score was calculated by summing scores from all three domains. Quartile-based classification was used for interpretation: for tribal women, a score > 24 was “Good,” >22 “Neutral,” and > 18 “Poor.” For adolescent girls, > 22 was “Good,” >21 “Neutral,” and > 13 “Poor.” For the mixed group, > 24 indicated “Good,” >23 “Neutral,” and > 20 “Poor.” The IQR highlighted score spread, showing that 75% scored above Q1, 50% above the median, and 25% above Q3. This approach helped identify knowledge gaps and prioritise areas for targeted interventions. Further details are in Table 3: Group-wise calculation and interpretation of KAP score.

**Table 3:** Group-wise calculation and interpretation of KAP score

Tribal Women group (20–49 Years) *N* = 355				Adolescent Girls (10–14 Years) *N* = 349			Mixed (15–19 Years) *N* = 43		
	Knowledge Score	Attitude Score	Practice Score	Knowledge Score	Attitude Score	Practice Score	Knowledge Score	Attitude Score	Practice Score
No of Questions	2	4	4	2	4	3	2	4	4
Mean	1.52	14.2	4.57	1.46	13.9	3.58	1.86	15.5	4.79
S.D.	0.851	2.26	2.37	0.891	2.46	1.97	0.516	2.68	1.36
Median	2	15	5	2	14	4	2	16	5
Q1/Q2/Q3	Q1 = 2 Q2 = 2 Q3 = 2	Q1 = 12 Q2 = 15 Q3 = 16	Q1 = 2 Q2 = 5 Q3 = 6	Q1 = 0 Q2 = 2 Q3 = 2	Q1 = 11 Q2 = 14 Q3 = 16	Q1 = 2 Q2 = 4 Q3 = 5	Q1 = 2 Q2 = 2 Q3 = 2	Q1 = 14 Q2 = 16 Q3 = 17	Q1 = 4 Q2 = 5 Q3 = 6
IQR	0.00	4.00	4.00	2.00	5.00	3.00	0.00	3.00	2.00
	Total KAP Score			Total KAP Score			Total KAP Score		
Mean Total KAP	20.28			18.92			22.16		
Total Median KAP	22			21			23		
Q1/Q2/Q3Total	Q1 = 18	Q2 = 22	Q3 = 24	Q1 = 13	Q2 = 21	Q3 = 22	Q1 = 20	Q2 = 23	Q3 = 24

## Discussion

**Fig. 2:**
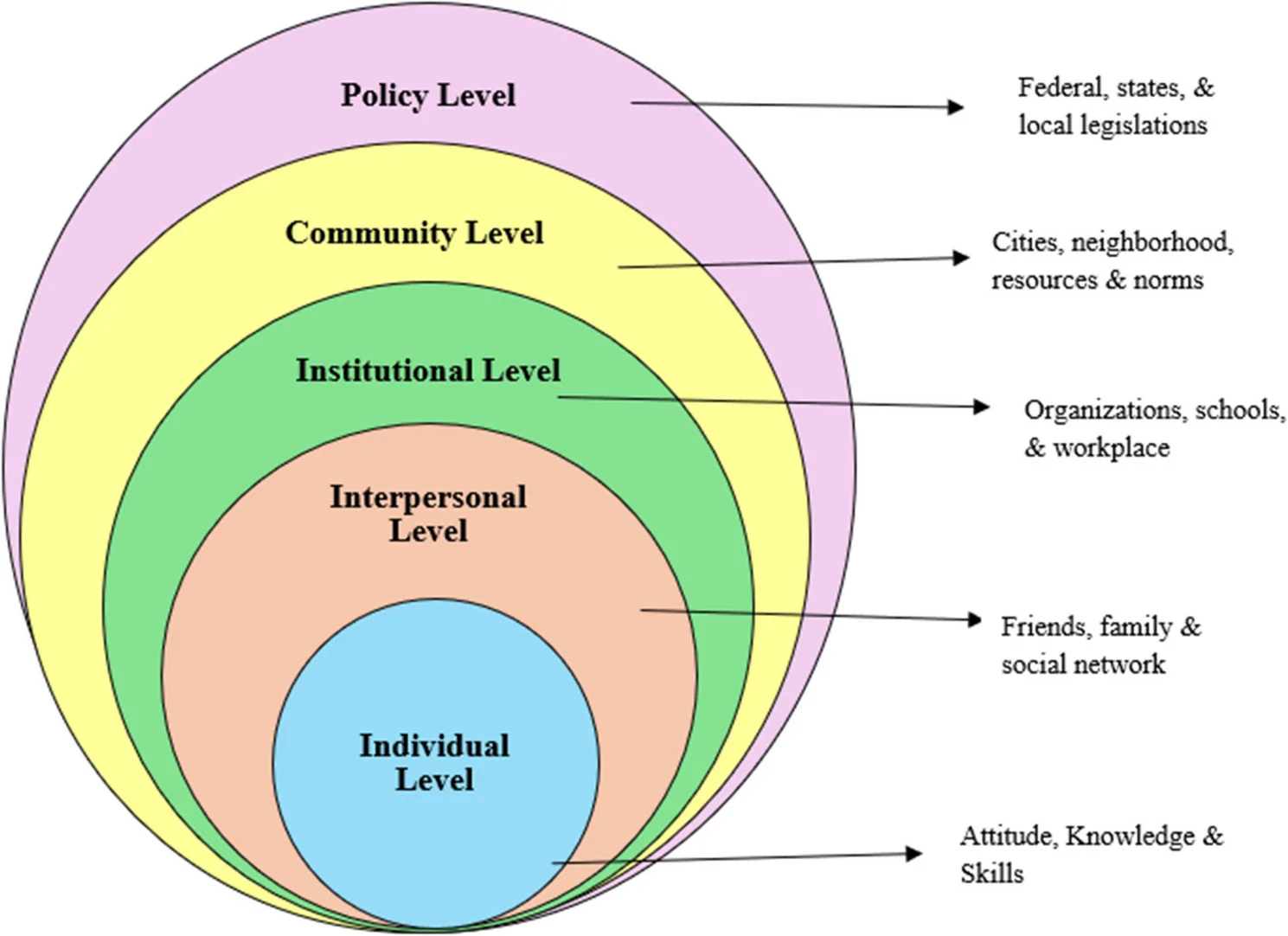
Socio-ecological model as a framework to understand the usage of healthcare schemes from tribal women in the reproductive age group

The current study highlights significant KAP gaps concerning reproductive health schemes among adolescent girls and tribal women, using the Socio-Ecological Model (SEM) framework illustrated in Fig. 2 [15]. This model offers a layered approach to examining the multiple influences: individual, interpersonal, community, institutional, and policy levels that affect awareness and participation in healthcare programs.

### Individual level

At the personal level, awareness was largely incomplete with tribal women in the reproductive age group (15–49 years) and adolescent girls (aged 10–14), demonstrating only partial knowledge of reproductive health schemes. The percentage of individuals with a comprehensive understanding was minimal in tribal women (15–49 years) and adolescents (10–14 years). Educational attainment was strongly associated with improved awareness and more positive attitudes, underscoring the critical role of literacy in effective health communication. Similar findings have been reported by other studies [16, 17], which found education to be a pivotal determinant of reproductive health knowledge in marginalised populations across India. Moreover, the appeal of schemes offering cash benefits aligns with previous research [18], where financial incentives significantly influenced healthcare participation. A similar study done in Egypt also stated that clinics under an incentive payment scheme for family planning services showed improvements in provider behaviour, such as better history-taking, more follow-ups, and more involvement of clients in contraceptive choice, indicating enhanced care quality [19].

### Interpersonal level

Information sharing through interpersonal relationships, particularly via family members, peers, and community health workers such as ASHA and AWW, was crucial in raising awareness. While tribal women predominantly received health information from ASHA workers, adolescent girls mostly obtained it through educational institutions, highlighting the importance of school-based interventions. Existing literature supports the idea that strong interpersonal connections can enhance healthcare-seeking behaviours [20, 21, 22]. A similar study from Zambia also stated that the teachers and community-based health workers (CBHWs) played pivotal roles in mobilising parents through school Parent-Teacher Association (PTA) meetings, countering misconceptions about adolescent reproductive health, addressing stigma around contraception, and facilitating adolescent-friendly discussions [23]. However, barriers like mistrust in the healthcare system, often stemming from bureaucratic inefficiencies and eligibility restrictions, negatively affect the uptake of these services. One such barrier identified in this study was the lack of bank accounts, which prevented participants from accessing financial benefits, consistent with earlier research on the Janani Suraksha Yojana [24, 25].

### Institutional level

Institutionally, disparities in benefit access were noted across age groups and tribal identity. While several tribal women accessed healthcare schemes, fewer women received financial assistance. Among adolescent girls, utilisation increased with the age group 15–19 years compared to the 10–14 years age group. suggesting that older adolescents are more engaged with the healthcare system. The similarly aspect was observed in a community-based cross-sectional study in Ethiopia, which found that older adolescents were significantly more likely to use reproductive health services than their younger peers [26]. Procedural inefficiencies, such as delays in financial disbursements and weak grievance mechanisms, remain obstacles, as seen in reports on PMMVY and JSY [27, 28, 29]. Streamlining these processes would not only boost efficiency but also improve community trust.

### Community level

At the community level, perceptions toward healthcare schemes were generally favourable, with more than half of respondents acknowledging the value of the schemes, financial incentives, and collective participation. Yet, endorsement of these schemes was relatively low among adolescent girls (10–14 years) and tribal women (15–49 years) actively recommended them. This hesitance mirrors findings by various studies, where rural populations expressed scepticism about the effectiveness of government schemes due to erratic implementation [30, 31]. Additionally, communication challenges such as language differences and culturally irrelevant messaging further limited outreach. Respondents proposed using more relatable and engaging methods like street plays, symbolic visuals, and messages in local dialects, recommendations that echo the effectiveness of culturally tailored strategies observed by other studies [32, 33].

### Policy level

At the policy level, focused action is needed to close the awareness and access gaps. Education emerged as a key factor across all KAP aspects, validating the importance of embedding reproductive health content in the school syllabus. One study has emphasised the value of structured, school-based health education [34]. Similarly, another study in rural Uganda showed that structured school-based programs among young adolescents (ages 11–15) improved Sexual Reproductive Health knowledge, behaviours, and attitudes, demonstrating the effectiveness of early, formal interventions [35]. Other factors, such as addressing eligibility-related issues through better financial inclusion strategies, such as simplifying banking procedures and reducing document requirements, could enhance scheme access. One study highlighted that the timely disbursement of incentives through bank linkages is critical, especially in underserved regions with limited banking infrastructure [36].

### Strength and limitations

The study’s strength lies in its integrated approach, combining quantitative and qualitative data from the semi-structured questionnaire. Quartile-based KAP score analysis offered a systematic understanding of awareness levels. However, limitations include possible selection bias. When participation is voluntary, individuals who choose to respond may systematically differ from those who don’t, perhaps being more motivated, interested, or having stronger opinions, thus limiting how well the sample represents the broader population. Individuals who consented to participate may have been more health-conscious, better informed, or more engaged with healthcare services than non-participants. This could have led to a potential overestimation of awareness and utilisation levels. Recall bias is another limitatio as asking participants to remember past experiences may cause inaccuracies due to flawed memory, especially in cross-sectional or retrospective designs. The study was limited to only one geographical region with the tribal community, especially among Santhals. The Particularly Vulnerable Tribal Groups (PVTG) were not included.

## Conclusion

The study reveals notable gaps in knowledge, attitudes, and practices regarding healthcare schemes among tribal women and adolescent girls, with educational status and tribal identity significantly influencing awareness and usage regarding reproductive health schemes among tribal women of Jharkhand. Overall findings indicate limited awareness, mixed attitudes, and gaps in the utilisation of available schemes. KAP scores highlighted knowledge deficits, and the majority had a positive attitude. However, the practices related to the utilisation of reproductive health schemes were found to be more inclined towards the schemes with cash incentives. Knowledge of major schemes (e.g., JSY, PMMVY, RKSK) remains insufficient, with substantial variation in awareness across tribal women, particularly lower among less-educated individuals. Attitude was positively influenced by age, educational level and exposure to interpersonal health communication. Yet practical uptake was hindered by systemic barriers such as financial inclusion issues and procedural delays. Disparities in service utilisation were particularly pronounced by educational status and tribal affiliation.

## Recommendations 

Enhancing cultural sensitivity through local languages, visuals, and community involvement, along with simplified processes and trust-building, is essential for improving outcomes.

## Supplementary Information

Supplementary Material 1

## Data Availability

Data will be available upon reasonable request.

## Abbreviations

NFHS: National Family Health Survey
IFA: Iron Folic Acid
SDG: Sustainable Development Goals
KAP: Knowledge, Attitude, Practices
ANC: Antenatal Care
JSY: Janani Suraksha Yojana
JSSK: Janani Shishu Suraksha Karyakram
PMMVY: Pradhan Mantri Matru Vandana Yojana
RKSK: Rashtriya Kishor Swasthya Karyakram
ASHA: Accredited Social Health Activist
CVI: Content Validity Index
AWW: Anganwadi Workers
IQR: Interquartile Range
SEM: Socio-Ecological Model
PVTG: Particularly Vulnerable Tribal Groups
