# Supplementary material for: Assessing the knowledge, attitude, and practices of reproductive health schemes among tribal women in Jharkhand: A cross-sectional study
Source: BMC Public Health. 2026 Jul 8;26:2721. doi: 10.1186/s12889-026-28411-z (PMC13628958; doi:10.1186/s12889-026-28411-z)
Supplement: Supplementary file 1 [file 12889_2026_28411_MOESM1_ESM.docx]

Questions validated by the external experts (HODs, Associate Professors, Assistant Professors, and Research Officers) for each question CVI-R index reported 0.9. Only two questions in the questionnaire for objective one reported CVI-R index 0.6(these questions have been refined according to the remarks by the validators)

**Title:** Development & Validation of a health awareness booklet: “Reproductive Health Schemes for tribal women of Jharkhand”

**Schemes included:**

| **Adolescent Girls schemes** | **Schemes for reproductive age group women** |
| --- | --- |
| 1.Rashtriya Kishori Swasthya Karyakram | 4.Janani Suraksha Yojana |
| 2.POSHAN Abhiyan | 5.Janani Sishu Suraksha Karyakaram |
| 3.Anaemia Mukt Bharat | 6.Pradhan Mantri Matri Vandana Yojana |
|  | 7.Pradhan Mantri Surakshit Matritva Abhiyan |
|  | 8. Family Planning Schemes under NHM including:    a. ASHA Scheme    b. Enhanced Compensatory Scheme |

**Permission from Tribal Welfare Officer will be taken after IEC Clearance.**

**Objective 1:** To assess the knowledge, attitude, and practices of healthcare schemes among reproductive age group tribal women.

| **Questions** | **Response** |
| --- | --- |
| **Socio-Demographic** | |
| Age | …........................ |
| Religion | - Hindu - Christian - Sarna Dharam - Other |
| Tribal Affiliation | -----------------(Specify name of the tribe) |
| Education | …........................ |

| Occupation | - Self employed - Daily wage workers - Government job - Private job - Other please specify |
| --- | --- |
| Marital status | - Unmarried - Married - Widowed - Divorcee |
| **Knowledge questions** | |
| Are you aware of the healthcare schemes mentioned above for the reproductive age  group women and adolescent girls. | - Yes - No - Partial Aware |
| If yes, please mention the name of the health  care schemes. | -------------- |
| Do you know the services provided under the  health care scheme/s? | - Yes - Somewhat - No |
| If yes, mention the services briefly. |  |
| Have you ever received any information or awareness campaign about the health schemes in your locality, Anganwadi centres, or through  the hospital? | - Yes - No |
| If yes, how did you receive the information | - Through ASHA (Accredited Social Health Activist) - Through AWW (Anganwadi Workers) - Family /friends - Posters - Television or radio - Community meetings - Others specify |
| Did you receive any kind of financial cash  assistance from the healthcare schemes? | - Yes - No |
| If yes, then how much money did you receive? | ------------- |
| In whose bank account the money got credited? | - Beneficiary account - Husband account - Parents account - In-laws account - Others |
| How was the incentivised money utilized?  Please specify | ------------------------- |
| **Attitude Questions** | |
| Do you think the program is beneficial in  improving healthcare services in your region? | - Strongly Agree - Somewhat Agree |

|  | - Neutral - Somewhat disagree - Strongly disagree |
| --- | --- |
| In your opinion, how important is it to receive the cash incentives through the program? | - Very Important - Somewhat important - Neutral - Not very important - Not important at all |
| How would you rate the overall experience with the Program where 1 is poor and 5 is excellent | - Excellent - Good - Fair - Average - Poor |
| Are you willing to participate in community health initiatives to improve the effectiveness of the health programs/schemes? | - Yes - No - May be |
| **Practices Questions** | |
| Would you recommend the scheme to other tribal women in your area? | - Yes - Unsure - No |
| Have you or your family members availed the benefits of these schemes? | - Yes - No |
| If you answered "Yes" to the previous question,  please provide details of your experience? | ----------------------------- |
| If you have not availed the benefits of the health programs/schemes, then what were the reasons for not utilizing the program? (Select all that apply) | - Lack of awareness - Did not meet the eligibility criteria - Too much time in the procedure - Lack of trust in the healthcare system - Other (please specify) |
| Do you think that the existing health programs/schemes are enough to meet the health care needs of the tribal community? | - Yes - No - Unsure |
| If no, then please provide some suggestions to  improve. | -------------------------------- |

**Objective 2:** To understand the challenges faced in provision of tribal women in the reproductive age group healthcare schemes from different Stakeholders perspective.

- Ice breaking questions and Self Introduction

1. From your experience, what aspect of the reproductive healthcare schemes have proven effective in improving the health of a tribal women?

**Probe: a**. key strengths of the current healthcare schemes for tribal women.

b. Governments commitment to tribal welfare, successful initiatives.
c. Training and capacity buildings.

2. What are the major weaknesses or gaps in the existing healthcare schemes targeted for reproductive age group tribal women?

**Probe: a.** Lack of infrastructure or accessibility issues hinderance in the implementation of these healthcare programs.

b. Any challenges related to cultural sensitivity or community engagement that impact the effectiveness of these schemes.
c. How do factors like language barriers and cultural difference affects patient care?
d. Primary health care service gaps, challenges in recruiting and retaining healthcare staffs, audits, **maintenance** of records.

3. How can community-based healthcare schemes empower tribal women in taking charge of their reproductive health?

**Probe: a. opportunities** exist to enhance local cultural practices and tradition to enhance the effectiveness of the schemes.

b. collaboration with NGOs, local leader support in awareness

c. technology such as mobile app, telemedicine can help in bridging the gaps in the remote area.

4. How can the lack of awareness and education about health care schemes of tribal women of reproductive age group pose a challenges to tribal women.

**Probe: a.** traditional beliefs and cultural norms pose challenges to the adoption of modern reproductive health care practices.

**Objective 3:** To develop & validate the awareness booklet for the tribal women of reproductive age group and evaluate the perception of participants towards it.

**Questionnaire for pre-post-test for developed booklet training sessions among the training participants:**

| **Questions** | **Response** |
| --- | --- |
| 1. What are the services covered under Janani Shishu Suraksha Karyakram? | - Free and cash less delivery - Free drop back from institution to home any time after delivery. - Free provision of blood to sick newborn till 90 days from birth - JSSK is only applicable for first pregnancy |
| 2. What is the cash incentive of pregnant women for rural area in low performing states under Janani Suraksha Yojana? | - Rs 700/- - Rs 1000/- - Rs 1400/- - Rs 600/- |
| 3. Is Last Menstrual Period (LMP) Date mandatory to be provided to claim benefits under the Pradhan Mantri Matri Vandana Yojana Scheme? | - Yes - No |
| 4. What are the benefits under the Pradhan Mantri Surakshit Matritva Abhiyan? | - Antenatal care services 9th of every month - PMSMA only provides routine check-ups and no specialized care. - Comprehensive and quality antenatal healthcare free of cost to first time pregnant women. - PMSMA is only for urban pregnant women |
| 5. How much is the cash incentives to the women undergoing for tubectomy at government facilities under Enhanced compensatory scheme? | - Rs 1200/- - Rs 1300/- - Rs 1400/- - Rs 1500/- |
| 6. What is the charge for home delivery of Oral Contraceptive Pills, by ASHA under National Family Planning “ASHA Scheme”? | - Rs 5/- for a cycle of OCP - Rs 1/- for a cycle of OCP - Rs 10/- for a cycle of OCP - Rs 4/- for a cycle of OCP |
| 7. Which is the benefit covered under Rastriya Kishori Swasthya Karyakram? | - Sanitary napkins - Mid-day meals - Only physical health and nutrition |

|  | - RKSK promotes early marriage and safe   motherhood |
| --- | --- |
| 8. What is the colour of the Iron Folic Acid Tablets? | - Red for Pregnant and lactating women - White for Pregnant and lactating women - Blue for Pregnant and lactating women - Orange for Pregnant and lactating women |
| 9. Deworming is done in: | - Women of age group 20-49 years Tri annually - Women of age group 20-49 with intestinal disease - Women of reproductive age group 20-49 years Annually - Women of reproductive age group 20-49 years Biannually |
| 10. Which of the option is a part of POSHAN Abhiyan for adolescents? | - POSHAN Abhiyan is only for infant and children - Delivery of supplementary nutrition at Anganwadi centres - POSHAN Abhiyaan is only about providing information and not practical interventions. - POSHAN Abhiyaan is not relevant for urban   areas or well-off families. |

**Feedback survey Questionnaire for participants after the training sessions about training session and contents in the booklet.**

| **Questions** | **Response** |
| --- | --- |
| 1. Rate the overall quality of the training sessions. | - Excellent - Good - Average - Below average - Poor |
| 2. You are satisfied with the training process? | - Strongly agree - Agree - Neutral - Disagree - Strongly disagree |
| 3. The training material was helpful in understanding the content. | - Strongly agree - Agree - Neutral - Disagree |

|  | - Strongly disagree |
| --- | --- |
| 4. The duration of training was appropriate for you. | - Strongly agree - Agree - Neutral - Disagree - Strongly disagree |
| 5. The pace of the training process was right for you. | - Strongly agree - Agree - Neutral - Disagree - Strongly disagree |
| 6. The content of the booklet was appropriate. | - Strongly agree - Agree - Neutral - Disagree - Strongly disagree |
| 7. The concepts presented clearly and effectively in simple understandable language. | - Strongly agree - Agree - Neutral - Disagree - Strongly disagree |
| 8. The tribal prints and designs in the booklet were attractive &representing the relevance. | - Strongly agree - Agree - Neutral - Disagree - Strongly disagree |
| 9.The message was effectively communicated through pictorial presentation in the booklet. | - Strongly agree - Agree - Neutral - Disagree - Strongly disagree |
| 10. The Audiovisual clips, and interactive sessions (Nukkad Natak & open Q&A session) in regional language helped you understand the health schemes. | - Strongly agree - Agree - Neutral - Disagree - Strongly disagree |
| 11. The instructor/trainers communicated the material effectively. | - Strongly agree - Agree - Neutral - Disagree - Strongly disagree |

| 12. The instructors were knowledgeable enough to answer your queries. | - Strongly agree - Agree - Neutral - Disagree - Strongly disagree |
| --- | --- |
